# The Mechanisms of CHD8 in Neurodevelopment and Autism Spectrum Disorders

**DOI:** 10.3390/genes12081133

**Published:** 2021-07-26

**Authors:** Orly Weissberg, Evan Elliott

**Affiliations:** Azrieli Faculty of Medicine, Bar Ilan University, Ramat Gan 13215, Israel; ov941908@gmail.com

**Keywords:** CHD8, autism, neurodevelopment, mouse model

## Abstract

Chromodomain-helicase-DNA-binding protein 8 (CHD8) has been identified as one of the genes with the strongest association with autism. The CHD8 protein is a transcriptional regulator that is expressed in nearly all cell types and has been implicated in multiple cellular processes, including cell cycle, cell adhesion, neuronal development, myelination, and synaptogenesis. Considering the central role of CHD8 in the genetics of autism, a deeper understanding of the physiological functions of CHD8 is important to understand the development of the autism phenotype and potential therapeutic targets. Different CHD8 mutant mouse models were developed to determine autism-like phenotypes and to fully understand their mechanisms. Here, we review the current knowledge on CHD8, with an emphasis on mechanistic lessons gained from animal models that have been studied.

## 1. Introduction to Autism

Autism spectrum disorder (ASD) is a heterogeneous neurodevelopmental disorder characterized by impaired sociability and language development, and repetitive and stereotypic behaviors. Autism has a strong genetic etiology, including involvement of chromatin rearrangements, de novo mutations, and common variants [1,2,3]. An interaction of multiple genetic factors and environmental factors may be involved in the development of ASD [4]. ASD incidence is steadily rising in the world population [5]. The prevalence is estimated to be one in 54 children at 8 years of age by the Centers for Disease Control and Prevention (CDC), or one in 40 at ages 3 to 17 years of age according to the National Survey of Children’s Health (NSCH) and the National Health Interview Survey. There is a male bias in the diagnosis of autism in the order of 4.3:1 in the USA [6]. The cost of treating ASD individuals including educational support, loss of parent working days, special health services, and others was USD 268 billion in 2015, and estimated to be USD 461 billion in 2025 in the USA [6,7,8]. The *Diagnostic and Statistical Manual of Mental Disorders,* Fifth Edition (DSM-5) defines autism using social communication impairment, restricted interests, and repetitive behaviors. Comorbidities include anxiety, epilepsy, attention deficit hyperactivity disorder (ADHD), speech and language delay, sleep disorders, gastrointestinal problems, and impaired learning and motor difficulties [9,10,11,12,13,14]. To date, there is no biological diagnostic assay [15,16,17] or approved curative treatment [18,19]. ASD etiology is poorly understood [20], and it is now accepted that it includes different subtypes induced by different etiologies and pathways, including genetic and environmental factors [12].

## 2. Association of CHD8 and Autism

Chromodomain-helicase-DNA-binding protein 8 (CHD8) was first linked to ASD about a decade ago, when de novo mutations were first identified in two ASD children (Table S1) [21]. Since then, many studies have shown that various disruptive mutations in both isoforms of CHD8 correlate with increased risk for ASD and might characterize an ASD subtype [22,23,24,25]. In Table S1, we summarize and describe the mutations that have been found to be associated with ASD. Most of the known CHD8 mutations lead to loss of function of the protein [26]. In ASD individuals, it was found that CHD8 mutations were more abundant in males [27]. In a cohort of ~6000 individuals with autism, 0.2% had de novo mutations specifically in CHD8 [28], further demonstrating that CHD8 dysfunction is an important factor in ASD pathology.

Individuals carrying CHD8 mutations exhibit a unique phenotype. One of the prominent features is significantly large head sizes (macrocephaly) that appears in ~85% of cases [29,30,31,32]. These individuals have typical face characterized by broad forehead, flat nasal bridge, large ears, increased occipitofrontal circumference (OFC), pronounced supraorbital brow ridges, and pointed chin [23,33]. Many of them are tall and slender (~47%) and have additional problems such as gastrointestinal disturbance (~40%) and sleep problems (~50%) [23,31]. In addition, they exhibit mild regression, attention deficit and anxiety [34,35], and developmental delay [36]. Speech delay, cognitive impairment, or intellectual disability (ID) appears in ~66–81% of cases. Additional comorbidities include motor deficits (hypotonia or excessive clumsiness) (~30%) [31,36] and seizures (27%) [31]. In this review, we first review the major studies that have looked into basic molecular functions of CHD8, and then review all major studies that have gained knowledge of its function in neurodevelopment and ASD through nonmammalian and mammalian animal studies.

## 3. Basic Molecular Functions of CHD8

CHD8 is located on 14q11.2. It is part of the SNF2H-like ATP-dependent chromatin remodeling enzymes family referred to as CHD (chromodomain helicase DNA binding) [37]. CHD8 has two isoforms: CHD8L, a full-length protein of 280 kDa; and CHD8S (Duplin), a 110 kDa protein of the NH2-terminal chromodomain region resulting from alternative splicing [38]. Despite having similar functions as other CHD family members, CHD8 needs higher nucleosome concentration for ATPase activation [39]. In initial studies, CHD8 was found to directly interact with β-catenin and negatively regulate its targeted genes [37], including Wnt signaling [40]. A separate study found that CHD8 promotes transcription of E2F target genes, thus regulating cell cycle [41]. Additionally, it negatively regulates p53 by recruiting histone H1, therefore suggesting a involvement in cancer-related processes [38]. CHD8 also interacts with CTCF. It was found that CHD8 knockdown induces a decrease in the expression of a subset of CTCF targets [42]. In addition, CHD8 interacts with CHD7, and together function in transcriptional regulation through RNA polymerase II [43]. These studies suggest a complex role for CHD8 in transcription regulation, where it may upregulate or downregulate transcription, depending on cofactors recruited and genomic regions targeted.

CHD8 has been found to be involved in many basic biological processes. RE-1 silencing transcription factor (REST), a transcription factor for neuronal genes, is regulated directly by CHD8. Reduced expression of CHD8 led to alterations in REST chromatin-binding and neurodevelopment deficits [22]. CHD8 stimulates histone H3 lysine 4 (H3K4) methylation through interaction with mixed-lineage leukemia (MLL), and influences oligodendrocyte maturation [44,45,46]. The neuromuscular junction (NMJ) structure and function is found to be maintained due to CHD8 regulation. In addition, CHD8 is involved in the cell cycle G1/S phase by binding to the promoters of cyclin E2 (ccne2) and thymidylate synthase (TYMS) [47].

In human neural progenitor cells (NPCs), human neuroprogenitor stem cells (hNPSC), and human neural stem cells (hNSCs), CHD8 was found to regulate the transcription of ASD risk factors [28] and brain-development pathways, including neuron differentiation and synapse development, cell adhesion, and axon guidance [44,48,49]. CHD8 is essential for development, as homozygote mutant mice die at an embryonic stage [38].

## 4. Expression of CHD8 and Knockdown of CHD8 in In Vitro or Nonmammalian Model Systems

**Figure 1:**
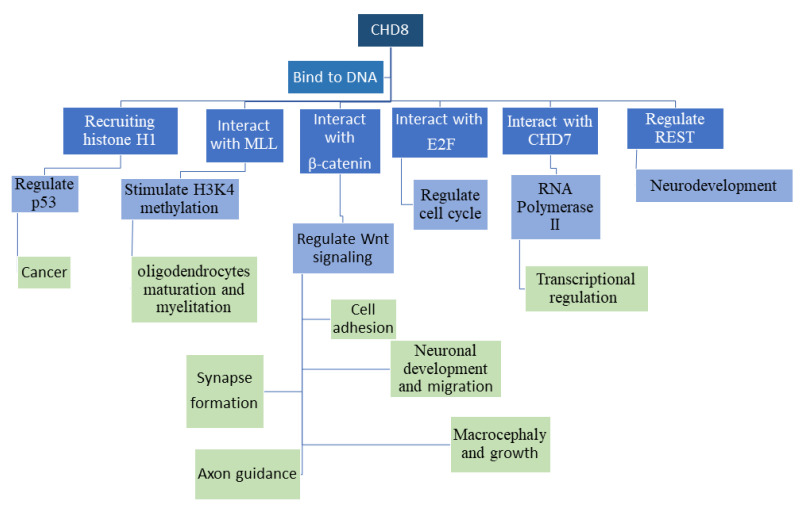
Functions of the CHD8 protein. CHD8 binds to DNA, and interacts with DNA binding proteins, leading to downstream changes in both epigenetic markers and downstream biological processes. This figure outlines some of the major interacting partners and downstream pathways that are regulated by each interaction.

CHD8 is expressed in the mouse at the embryonic stage (E12.5) in different levels in wide regions of the brain (neocortex, forebrain, ventricular, subventricular and mantle zones, rhombic lip (RL), and the isthmus of the cerebellum, as well as in lower RL and floor plate region of the hindbrain, midbrain, diencephalon, hypothalamus, pituitary gland, craniofacial region, and tongue and olfactory epithelium). In the postnatal mouse brain (P20), CHD8 is expressed in the cerebellum, neocortex, hippocampus, hypothalamus, and olfactory bulb [50]. Peak expression levels were observed at E18-P7, then gradually decreased to adulthood. Highest expression was found in neurons, and lower levels in astrocyte and astroglia [51]. In the mouse, CHD8 is expressed higher in brain compared to other tissues and in the embryo, compared to adult [52]. CHD8, as a chromatin remodeling protein, affects a wide variety of signaling and biological functions (Figure 1), as will be expounded upon throughout this review, and therefore can be crucial in ADS etiology.

Several studies have looked into the role of embryonic CHD8 in neuronal development. Durack et al. used in vivo electroporation to introduce shRNA targeting CHD8 in fetal cerebral cortex [47]. Using this method, Xu et al. found that CHD8 is crucial to axon and dendritic growth and development. In addition, CHD8 is essential to neuronal migration in the cortex at the embryonic stage, although it can recover after birth [51]. Reducing CHD8 expression in upper layer cortical neurons at this time point induced social behavior deficits and anxiety in the adult offspring. CHD8 shRNA knockdown induced a decrease in neuronal precursor proliferation, concurrent with a decrease in cell-cycle genes and an increase in genes involved in neuronal proliferation. They further showed that CHD8 promotes neuronal precursor proliferation through the stimulation of the Wnt signaling pathway [47]. In a separate study, RNA-seq of CHD8 +/− cerebral organoids, generated from induced pluripotent stem cells (iPSCs) from skin fibroblasts, also revealed alteration of genes involved in neurogenesis, neuronal differentiation, forebrain development, and Wnt/β-catenin signaling [53]. Sood et al. induced mutation in the CHD8 exon 12 by CRISPR-Cas9 on mouse embryonic stem cells (ESCs) differentiated into NPCs, and found that CHD8 is important for NPC development and neurogenesis, as well as Wnt signaling and core pluripotency network OSKM. Additionally, they found CHD8’s effects as a transcriptional regulator are dosage dependent [54]. This emphasizes the importance of maintenance of correct CHD8 levels. A further study found that individuals with CHD8 mutation have unique DNA methylation (DNAm) that distinguishes them from neurotypical and from other ASD patients. This support the idea that CHD8 mutations create distinct ASD subtypes [55]. A mutation in genes parallel to CHD8 in Caenorhabditis elegans also induced neurological phenotypes, including locomotion and motor coordination defects, fecundity defects and length, width and body area alteration [56]. In Drosophila, CHD8 was found to interact with RIMS1 and involved in presynaptic homeostatic plasticity (PHP) and stability of synaptic transmission [57]. Therefore, CHD8 regulates neuronal development in several model organisms in similar pathways, particularly in pathways involving regulation of Wnt pathway.

Coll-Tané et al. [58] used drosophila to reveal a possible mechanism in which CHD8 influences sleep. Interestingly, CHD8 mutant individuals often report difficulties in sleeping. Glia-specific knockout of kismet, the drosophila ortholog of CHD7 and CHD8, induced sleep disturbances. Furthermore, kismet regulated synaptic development in neurons. During development, high serotonin levels in glia cells near the blood–brain barrier are linked to sleep problems. The sleep disturbances were dependent on increases in serotonin levels during development. Therefore, this study found a specific role for glial CHD8 in sleep regulation.

## 5. Effects of CHD8 on Non-Neuronal Tissues

Several other studies have shown a role for CHD8 regulation in non-neuronal cells in both behavioral and physiological pathways. Slenderness is one of symptoms seen in individuals with CHD8 mutation. Kita et al. determined that CHD8 deletion specifically in preadipocytes leads to decrease in adipogenesis and a slender phenotype in mice. They found that CHD8 interaction with C/EBPα and PPARɤ is crucial for adipogenesis and increase of white adipose tissue mass. Therefore, CHD8 mutations may lead to physiological changes through regulation in peripheral tissues [59]. Separate studies found that oligodendrocyte precursor cell (OPC) proliferation, differentiation, and survival is regulated by CHD8 and CHD7. CHD8 interacts in OPCs with ASD risk factor genes, therefore it is suggested that oligodendrocytes take part in ASD pathology [60]. In oligodendrocytes from Chd8 OPC knockout mice (Chd8flox/flox; Olig1-Cre+), mutants displayed dysregulation in OPC differentiation, proliferation, and myelination. By recruiting KMT2 histone methyltransferase and H3K4, CHD8 regulates the BRG1-CHD7 cascade (BRG1activate CHD7) that is responsible for oligodendrocyte development. In addition, these mice die at P21, which is the peak time of myelination. Hence, it suggested that CHD8 is crucial for myelination regulation and OPC differentiation. In addition, GO analysis revealed CHD8 influences neurogenesis, gliogenesis, Wnt signaling, and apoptosis [45]. CHD8 inhibition resulted in macrocephaly [48] due to increase of forebrain and midbrain and impairment of postmitotic enteric neuron results in gastrointestinal dysfunction in zebrafish [23].

## 6. Mouse Models in the Research of CHD8, Neurodevelopment, and Autism

Various mouse models were developed to study CHD8 mutations in ASD. In Table S2, we have gathered and described the major mouse models produced to date. Each model used different techniques and often found slightly different characteristics. Here, we will review the different models and their findings in the aspects of transcriptome, brain development, and behavior (Table S2). Katayama et al. [22] generated two lines: Chd8+/ΔL by deletion of exons 12–14 (“ΔL”), therefore inducing a knockout only of the long isoform; and Chd8+/ΔSL by deletion of exons 2–10 (“ΔSL”), therefore inducing a complete knockout of both isoforms of CHD8. The different knockouts both produced prenatal lethality, demonstrating that CHD8 is necessary for vitality. However, haploinsufficient mice could be produced from both lines. Interestingly, haploinsufficiency of the large isoform had the same behavioral effect as the haploinsufficiency of both isoforms. Chd8+/ΔL mice demonstrated increased anxiety, repetitive behavior, and altered social behavior, in addition to neurodevelopmental delay in the embryos. These mice had macrocephaly, without changes in overall body weight, and demonstrated alterations in the expression of embryonic neurodevelopmental genes in the brain. Mutant mice also have shorter intestines and slow intestinal transit, similar to gastrointestinal problems in autism.

In a separate study, Cherepanov et al. [61] used the CHD8+/∆SL mice to test sex-specific behavior differences and the possible role of oxytocin in the behavioral phenotype. They found anxiogenic behavior in both males and female CHD8+/∆SL mice, while depressive phenotype appeared only in CHD8+/∆SL female mice. Locomotion, social avoidance, and sociability were normal in CHD8+/∆SL mice compared to WT. Social preference toward a novel mouse was impaired in CHD8+/∆SL male mice, while females had increased to novelty. Of interest, oxytocin concentration in plasma decreased specifically in CHD8+/∆SL male mice. Peripheral administration of oxytocin improved anxiety only in males, but normal social novelty behavior was recovered in both sexes. Therefore, oxytocin levels may play a direct role in behavioral abnormalities in this model.

Platt et al. [62] generated CHD8 haploinsufficiency (Chd8+/−) mice using a 7-nucleotide deletion in exon 1 that causes a frameshift mutation leading loss-of-function (LOF) that was found in ASD patients. Haploinsufficient Chd8 mice exhibited an autism-like phenotype including typical autistic behavior, macrocephaly, and craniofacial abnormalities similar to ASD individuals. In addition, they displayed anxiety, and mild impaired social interactions at P23–25. Interaction duration of juvenile social play was increased, while their number did not change. Reciprocal play behaviors did not change in number or duration. Repetitive behavior as observed in self-grooming events and the marble-burying test did not change. The three-chambered social approach task showed impaired social novelty, although sociability did not change. In adult mice, memory, as tested by contextual or toned fear-conditioning task, did not show changes. In open-field tests and dark–light emergence tests, mutant mice exhibited anxiety-like behaviors. Rotarod test determined acquired motor learning was increased. These mice displayed lower body size and showed alterations of adulthood brain gene expression in genes implicated in DNA modification, mRNA and protein formation, Wnt pathway, and cell-cycle functions. Cortical progenitor cell population of the somatosensory cortex was increased in E15.5 mice. MRI analysis determined that CHD8+/− mice brain volume and intraocular distance were larger, in comparison to wild-type controls. In addition, they showed striatal dysfunction. Inhibitory synaptic transmission in adult mice found no differences in frequency of miniature inhibitory postsynaptic current (mIPSC); however, a decrease in their amplitude was found. Additionally, during adulthood, electrophysiology of medium spiny neurons (MSNs) from the nucleus accumbens revealed synaptic changes. Therefore, in this model, various autism-related abnormalities were observed in the juvenile period, and motor learning in a specific striatal pathway was dysregulated in adulthood.

Gompers et al. [63] created the Chd8+/del5 mouse strain by germline 5 bp deletion in Chd8 exon 5, upstream of the known human mutations. Chd8+/del5 mice displayed cognitive impairment in learning and memory in contextual and cued conditioning and novel object recognition. Chd8+/del5 mice had normal sociability and no repetitive behaviors. In Chd8+/del5 mice, transcriptional changes were found in neurodevelopmental disorder pathways such as neurogenesis, synaptic processes, and neuroimmune signaling. RNA-Seq analysis from Chd8+/del5 mice during development (E12.5, E14.5, E17.5, P0) and adulthood exhibited increased expression in genes involved in neural progenitor proliferation, which may explain macrocephaly. Indeed, there was increased regional brain volume found in Chd8+/del5 mice. Cortical anteroposterior and neocortical regions were larger by ~7% and ~8%, respectively, at birth (P0). However, cortical thickness did not show changes. Additional areas that exhibited increased size in the brain included cerebral white matter (5.4%), cerebral gray matter (6.1%), cortex, hippocampus (10.3%), and amygdala (11.0%). A particularly novel finding was a dysregulation in RNA splicing in neurodevelopment-related genes in the Chd8+/del5 mice.

Jung et al. [52] created the Chd8+/N237k model using Asn2373LysfsX2 mutation (Asn2371LysfsX2 in human) in exon 37. Chd8+/N2373K mice displayed sex-dependent behavioral, molecular, and electrophysiological differences. In the maternal-separation paradigm, Chd8+/N2373K male pups (P5–11) showed increased mother-seeking behaviors (ultrasonic vocalizations (USVs) were faster, longer, and more frequent), while female pups were normal. In juvenile mice, open-field locomotion, play, or repetitive behaviors were not significantly dysregulated in male and females, although males showed a tendency toward hypoactivity. In adulthood, isolation-induced self-grooming was higher in adult male Chd8+/N2373K mice. As sex differences were found in behavior, all subsequent analysis was performed on both males and females. RNA-Seq of Chd8+/N2373K mice whole brain at P25 revealed three differentially expressed genes in males and 96 in females, although no differences were found at P0. The female-specific genes were related to extracellular vesicles, including blood microparticles and extracellular exosomes (genes for intercellular communication) and with the immune system. In female Chd8+/N2373K mice, downregulated genes were enriched for CHD8-binding genes. Using a network-based analysis to identify networks of genes that were changed in the mouse model, they determined that P0 male and female Chd8+/N2373K brains displayed transcriptomic patterns that partly mimicked those observed in human ASDs. Male Chd8+/N2373K mice at P25 showed a transcriptomic pattern that mimicked that of human ASD, whereas females showed a distinct and partly opposite pattern. Chd8+/N2373K mice birth rate and body weight remained normal. In order to determine changes in neuronal activity after environmental stress, C-fos levels were measured in mouse models at basal conditions and after maternal separation. At basal conditions, Chd8+/N2373K female mice had significantly lower levels of c-fos signal in cortical and subcortical regions. Male levels were the same as WT mice. However, mutant males separated from their mothers exhibited increased c-fos levels in different brain regions (such as the cortex, basal forebrain, hippocampus, and amygdala), despite decreased c-fos levels found in lateral habenula (LH). Female knockouts were not differentially affected by the maternal separation. Using electrophysiological analysis, it was found that neuronal activity increased in Chd8+/N2373K brain in response to mother separation in males and females, most notably in the hippocampus, but also in the cortex, especially the prefrontal and sensory cortex. In the hippocampus, sexual differences in synaptic transmission were observed. The mIPSC frequency and amplitude were lower in males, whereas in females, its frequency was increased. Excitatory neuronal firing was also increased in mutant males, while in females, the inhibitory neuronal firing was increased. These studies demonstrate that a CHD8 mutation may affect males and females in very different ways at the molecular, electrophysiological, and behavioral levels.

Another CHD8 haploinsufficiency model was created by Suetterlin et al. [64]. CHD8+/− mice pups displayed hyperactivity and delayed motor and reflex function. In adulthood, the CHD8+/− mice showed more sociability and socio-communicative behavior in the three-chamber sociability test and hypoactivity in the open-field test. CHD8+/− mice had no anxiety in light/dark box test, and no differences in repetitive behavior in marble-burying and self-grooming tests. Spatial learning abilities and cognitive flexibility as measured by the Morris water maze test showed normal learning, normal cognition, spatial learning abilities, and flexibility. In addition, these mice exhibited normal behavior in USVs. Body growth was delayed from birth to P35; however, brain volume was increased up to 20.4% on P35. Furthermore, open-field activity was negatively correlated with hippocampal and cortical volume. Chd8+/− displayed dysregulation of the cortical transcriptome during early developmental stages. RNA-seq analysis of Chd8+/− mice from the neocortex at E12.5 and P5 revealed only five genes changed at E12.5, compared to 649 genes at P5 (66% of them were downregulated), when 56 of them are known as ASD-associated (almost all downregulated). KEGG pathways that are involved in protein transport, the ribosome, and oxidative phosphorylation were upregulated, while genes involved in cell adhesion, axon growth and guidance, and synaptogenesis were downregulated. Suz12 target genes were downregulated, which impacted histone methyl transferase and gene-silencing activities of Polycomb repressor complex 2 (PRC2). Long-range connectivity tested using rsfMRI showed functional overconnectivity between sensory regions of the neocortex and limbic cortical, auditory cortex, and ventral hippocampus regions. In summary, these Chd8+/− mice had macrocephaly and dysregulation of the cortical transcriptome, with behavioral changes that focused on changes in activity and motor function.

The Chd8V986*/+ mouse model [27] was generated in C57BL/6J blastocysts using CRISPR/Cas9. In this model, mutation p.Val984X was generated at the equivalent position in mouse Chd8 (V986), creating tandem stop codons. Pup survival was reduced in wild-type x Chd8V986*/+ crosses when the mutant parent was female. Chd8V986*/+ mutants exhibited decreases in rearing responses and center time in the open field, and increase in social novelty preference. At 1 year old, behavior abnormalities were more significant. Males at 6 months exhibited normal performance in the elevated plus-maze, marble-burying test, buried-food test, and acoustic-startle test. In addition, at 6 months, rearing movements were decreased, and this was more distinct at 1 year old. Open-field tests and social tests differed at 6 months but were severe at 1 year old. At 6 months and at 1 year old, open-field-test locomotor activity was similar, while social interactions and social novelty were increased. Chd8V986*/+ mice displayed at the embryonic stage (E14.5) transcriptomic alterations in pathways related to the maintenance of the excitatory–inhibitory balance, synaptogenesis, and neuronal maturation (decrease in genes associated with synaptic and neuronal function, sodium channel activity), which recovered to normal levels in the postnatal period. In adult mutant mice, there was impairment in proteostasis (decrease in genes associated with endoplasmic reticulum (ER) stress, chaperone-mediated protein folding, and the unfolded protein response (UPR) genes), and increase in the c-MET signaling pathway [26,27]. Cerebral cortex RNA-Seq at embryonic (E14.5) and postnatal ages (1, 6, and 12 months) revealed Chd8 expression was highest at E14.5 and persisted at a lower level throughout life. Therefore, of interest, this model displayed transcriptomic differences at early developmental periods, but major behavioral deficits were only detected at later stages of life.

In order to determine the role of CHD8 in oligodendrocytes, Olig1-Cre/Chd8F/F mice [65] were generated by crossing mice homozygous for a floxed Chd8L allele (Chd8L F/F Olig1-Cre/Chd8L mice) with mice that expressed Cre recombinase under the control of the mouse Olig1 promoter (Olig1-Cre mice). This created a mouse model of CHD8 deletion specifically in oligodendrocytes. Most of the pups died before 3 weeks of age, also displaying tremors and paralysis of the hind limbs. Therefore, they focused on haploinsufficiency. In Olig1-Cre/Chd8L+/F mice, decrease of CHD8 in oligodendrocytes led to abnormal behavior in mice, as expressed in increased social interaction and anxiety-like behavior. Olig1-Cre/Chd8L+/F mice had normal body and brain weight, as well as intestine length or intestinal transit, suggesting that macrocephaly and gastrointestinal defects are not induced through oligodendrocyte defects. Open-field tests differed in vertical activity, but not in total distance traveled and time spent. T-maze forced alternation tests, T-maze left–right discrimination tests, acoustic-startle response, and prepulse inhibition (PPI) did not differ, thus acoustic-startle response, learning and memory were normal. Anxiety-like behavior was tested by light–dark transition test, and light chamber time spent and distance traveled, as well as transitions between the light and dark chambers, were significantly decreased in mutant mice, which indicated increased anxiety-like behavior. CHD8 haploinsufficiency in oligodendrocytes caused anxiety-like behavior. Social interaction showed no differences in social contacts; however, total contact time was increased, and mild deficit in social novelty was observed. No difference was found in motility. Other behavioral tests, including the elevated plus-maze test, nest-building test, and Porsolt forced-swim test did not reveal significant differences. Olig1-Cre/Chd8LF/F mice had defective myelin formation and oligodendrocyte proliferation in the corpus callosum, as well as decrease of myelin basic protein (MBP) expression in the whole brain, corpus callosum, cerebellum, and spinal cord (P7 and P14). Olig1-Cre/Chd8L+/F mice had higher g-ratio and a thinner myelin sheath, as well as nodal widening and latency of CAP transmission, while the conduction velocity decreased in the corpus callosum. Pyramidal neurons in layers 2/3 of the prelimbic cortex showed no differences in the amplitude or frequency of spontaneous excitatory (sEPSC) or spontaneous inhibitory (sIPSC) postsynaptic (either Chd8+/∆L or Olig1-Cre/Chd8L+/F). EPSC, IPSC, and postsynaptic properties of pyramidal neurons in the prelimbic cortex did not significantly change in Olig1-Cre/Chd8L+/F mice. Therefore, CHD8 in oligodendrocytes has an important role in regulating myelination.

Hulbert et al. [66] generated a mouse model using a gene trap inserted after exon 31. Chd8+/E31T mice displayed normal anxiety-like behavior, repetitive self-grooming, learning impairments, and social interaction. Social novelty was also normal in three-chamber tests. Altered communication was observed in USV tests, as the mice exhibited an increase in call length. Chd8+/E31T mice had improved motor function rotarod task and had normal body weight, although they did exhibit increased brain size.

In a very recent study, Kawamura et al. [67] performed specific knockout of CHD8 in cerebellar granule neuron precursors, using the Atoh-promoter-driven Cre line. These mice displayed decreases in the proliferation of precursor cells and decreases in neuronal differentiation. Electrophysiology studies determined a decrease in both presynaptic and postsynaptic functions of the cerebellar granule neurons. In addition, the knockout mice displayed significant motor deficits, while not displaying any deficits in social interaction. Of interest, knockout of CHD8 in cerebellar perkinje cells did not give any of the above phenotypes. Therefore, CHD8 in cerebellar granule cells specifically regulates motor phenotypes.

## 7. Interplay between CHD8 and Other Genetic or Environmental Factors

As explained above, there is a very complex genetic etiology of autism, in addition to the possible interaction between genetics and environment in the development of ASD. Therefore, it is important to consider the possible interplay between CHD8 and these genetic and environmental factors in the development of ASD. One study found relatively high penetrance, approximately 50% for CHD8 mutations in ASD [32]. This is considerably higher than most other autism-associated genes, which suggests that CHD8 mutations may often be enough to induce the autism phenotype. However, since 50% of cases with CHD8 mutations did not display autism, this suggests additional genetic or environmental factors. Of great interest, this same study found that CHD8 mutations had a higher penetrance in males compared to females. While autism does have a male bias, not all autism-associated genes display such a bias. For example, Phelan-McDermid syndrome, characterized by deletion of genetic regions, including the gene Shank3, does not display higher penetrance among males [68]. Therefore, CHD8 specifically appears to have a sex-dependent effect. This is further verified by the mouse studies reviewed above, which showed sex-dependent effects of CHD8 deletion. Therefore, sex may be one genetic/biological factor that interacts with CHD8 in the development of ASD.

Chromatin-binding proteins are highly enriched in the known ASD-associated gene list [69]. Chromatin-binding proteins, including CHD8, often function as complexes. CHD8 interacts with the chromatin organizer CTCF [42], which has also been implicated in the genetics of ASD [70]. Together they regulate important regions in the genome, including the differentially methylated region (DMR) of H19, b-globin, BRCA1, and c-myc genes. Individuals with mutations in CTCF or CHD8 often show similar phenotypes, including speech delays and impaired social behavior, although there are some important differences as well (microcephaly vs. macrocephaly). More research is needed to understand the functional importance of the interaction between these two proteins. While many chromatin-binding factors have been associated with ASD, there is still little knowledge about which of these factors may directly bind and regulate CHD8 activity. This is an important question moving forward.

CHD8 has direct effects on biological pathways that have previously been implicated in autism. Therefore, if these pathways are otherwise perturbed, either through genetic or environmental means, then this may play a role in the development of ASD. In particular, CHD8 regulates the activity of the Wnt pathway. As discussed above, CHD8 binds to the promoter of Wnt signaling genes, including Fzd1, Dvl3, and β-catenin by binding to their promotor. CHD8 cortical knockdown led to decrease of TCF/LEF transcription factor family and impaired brain development [2]. Wnt-related genes have been implicated in ASD. In addition, Wnt pathways are important development-related pathways that can be disturbed by environmental factors. Therefore, an interaction between CHD8 mutations and the Wnt pathway may be important in the development of ASD.

## 8. Future

CHD8 is expressed in various cell types; however, it has cell-type-specific functions, as seen in the models described above [71]. It is expressed in higher levels during the embryonic and postnatal stages, hence it is important for development [51]. Many studies had emphasized CHD8 involvement in ASD in a way that it might be considered a separate ASD subtype. However, multiple questions remain open and need further study. The differential behavioral phenotypes seen in the models discussed above further suggest that different mutations may have separate effects on CHD8 function. The different phenotypes may be partly due to laboratory conditions, but are also likely due to disruption of different areas of the CHD8 gene. In addition, it is not clear if the role of CHD8 in neuronal activity is completely developmental, or if there are specific roles for CHD8 in brain function past development. In order to answer these questions, further study is needed.

## Supplementary Materials

The following are available online at https://www.mdpi.com/article/10.3390/genes12081133/s1, Table S1: known autism mutations, Table S2: CHD8 mouse models.
