# Supplementary material for: The Mechanisms of CHD8 in Neurodevelopment and Autism Spectrum Disorders
Source: Genes (Basel). 2021 Jul 26;12(8):1133. doi: 10.3390/genes12081133 (PMC8393912; doi:10.3390/genes12081133)
Supplement: Supplementary file 1 [file genes-12-01133-s001.zip › supplementary table 1.pdf]

Supplementary Table S1: known CHD8 mutations.

	**AA change**	S.M	Chr14 P	**NVIQ**	**VIQ**	**FSIQ**	INT	Mut	Phen
O'Roak et al. 2012 (1)	p.Gln959TERM			34			DN	n/a	ASD
Brian J. O'Roak et al. 2012 (2)	p.Ser62X	c.185C>G	21899618	78			DN	Ns	ASD
	p.Tyr747X		21878133	38			DN	Fs	ASD+ID
		c.3519-2A>G		47	37	43	DN	Sp	ASD
	p.Gln1238X	c.3712C>T	21871178	34	75	74	DN	Ns	ASD+ID
	p.Arg1337X	c.4009C > T	21870169	92			DN	Ns	ASD
	p.Glu2103ArgfsX3	c.6307_6310del	21861643	67			DN	Fs	ASD
	p.Leu2120ProfsX13	c.6359_6360del	21861376	93			DN	Fs	ASD
	p.Asn2371LysfsX2	c.7112_7113insA	21859175	19			DN	Fs	ASD+ID
	p.His2498del			98			DN	Aa	ASD
Bernier et al. 2014 (3)	p.Val984X						IN	Fs	ASD/ID
	p.Glu1114X	c.3340G>T		41	27	34	DN	Ns	ASD
	p.Glu1932SerfsX3						DN	Fs	ASD/ID/DD
	p.Glu2136ArgfsX6					<40	DN	Fs	ID
	p.Lys2287 del						n/a	Aa	ID/ADHD
	p.Arg910Gln	c.2729G>A			27		DN	Mns	ASD+ID
	p.Gly1710Val	c.5129G>T					IN	Mns	ASD+DD
	p.Arg1797Gln	c.5390G>A					IN	Mns	ASD
Prontera et al. 2014 (4)		~114Kb microdeletion	21,823,852 – 21,937,621		76		DN	Md	ASD
B. J. O'Roak et al. 2014 (5) (Supplementary)	p.Arg212Gln			72			DN	Ms	ASD
	p.Gln696Lys			125	88		DN	Ms	
	p.Met904Ile			63			DN	Ms	ASD+ID
	p.Arg1834X	c.5500C>T		93			DN	Ns	ASD+ID
	p.Arg1580Trp			74	97		DN	Ms	ASD
		c.4818-2A>C		103	96		DN	Ns	ASD
		c.5051 + 2T>A					DN	Ns	ASD

Iossifov et al. 2014 (6)		c.1593_1601_38del					DN	Ssv	ASD
De Rubeis et al. 2014 (7)	p.Leu834Pro						DN	Ms	ASD
	p.Arg1242Gln						DN	Ms	ASD
	p.Gly1602ValfsX15						DN	Ssv-Fs	ASD
	p.Ser1606ArgfsX8						DN	Ssv-Fs	ASD
	p.Tyr1642LeufsX25						DN	Ssv-Fs	ASD
Talkowski et al. 2012 (8)		t.14q11.2; 3q25.31					DN	Tr	ASD+ID
McCarthy et al. 2014 (9)	p.Ser2173X	c.6518C>A	21860919				DN	Ns	ASD+SHZ
Kimura et al. 2016 (10)	p.His1439del				50		DN		SCZ
	p.Lys2287del						DN		SCZ
	p.Arg2333Cys				50		DN		ASD+ID+DD+SCZ
	p.Arg773Gln						DN		ASD+SCZ
Merner et al. 2016 (11)	p.Asn2092LysfsX2	c.6276dup	21859176		56		DN	Fs	ASD+ID
	p.Arg7Cys	c.19C>T	21897482				IN	Pms	ASD
	p.Ile1325Thr	c.3974T>C	21868146				IN	nSn	SCZ
	p.Glu1750Lys	5248G>A	21861869				n/a	nSn	ASD
	p.Arg1879Cys	c.5635C>T	21860965				IN	nSn	SCZ
	p.Arg1901Cys	c.5701C>T	21860899				n/a	nSn	SCZ
	p.Gly1998Ala	c.5993G>C	21860047				n/a	nSn	ID
	p.Arg2035Gln	c.6104G>A	21859746				IN	nSn	SCZ
	p.Gly162Gly	G/C	21896306				IN	Sn	ASD
	p.Leu1305Leu	A/G	21868205				IN	Sn	SCZ
	p.Ala1693Ala	A/G	21862038				IN	Sn	SCZ
	p.Glu1825Glu	G/A	21861642				IN	Sn	ASD
	p.His1989His	C/T	21860073				IN	Sn	ID
	p.Asp2261Asp	C/T	21853898				IN	Sn	ASD

Smyk et al. 2016 (12)		~445 kb microduplication	21,507,092-21,952,439				DN	Md	DD+HDAD
Zahir et al. 2007 (13)		~101 kb microduplication	20,896,740 – 20,998,178				DN	Md	DD+ID
		~1.6 Mb microduplication	19,584,863 – 21,207,935				DN	Md	DD+ID
		~1.079 Mb microduplication	19,853,310 – 20,932,827				DN	Md	DD+ID
Terrone et al. 2014 (14)		~2.89 Mb microduplication	chr14: 19,788,445 - 22,675,219				DN	Md	ASD
T. Wang et al. 2016 (15)	p.Asp691GIy						DN	Ms	ASD
	p.Lys750AsnfsX14						DN	LGD	ASD
	p.Asn1235MetfsX18						DN	LGD	ASD
	p.Arg1897ThrfsX23		21862265				DN	LGD	ASD
Stolerman et al. 2016 (16)		Exons 26-28 deletion	21,863,796-21,868,103				DN		ASD+DD+ID
Han et al. 2018 (17)	p.Arg1551Cys	c.4651C>T		65			DN	Ms	ID
J. Wang et al. 2018 (18)	p.Glu883X	c.2647C > A					DN	Ns	ASD+DD
	p.Met559Ile	c.1677C > A					DN	Ms	ASD+DD
D'Gama et al. 2015 (19)	p.Val744Ile	c.2230G > A					DN	Ms	ASD+ID
Cappi et al. 2016 (20)	p.Elu1327Lys	C > T	21870199				DN	Ms	ID+OCD
Arnett et al. 2018 (21)	p.Glu1727X	c.5179G > T					DN	Sg	ASD
	p.Arg1402X	c.4204C > T					DN	Sg	ASD
	p.Ile1108AsnfsX7	c.3322_3323insA					DN	Fs	ASD
	p.Asn807ThrfsX78	c.2420del					DN	Fs	ASD
	p.Arg952X	c.2854C > T					DN	Sg	ASD

	p.His782ProfsX7	c.2345del					DN	Fs	ASD
		c.3882 + 1G > A					DN	Sp	ASD
Wong et al. 2019 (22)	p.Pro165Leu	c.494C>T					DN	Ms	ASD
Yasin et al. 2019 (23)		~33.269 kb deletion	21,827,942-21,861,211				DN		ASD+ID+DD
Ostrowski et al. 2019 (24)		c.470del					DN	Fs	ID
		c.517_533del					DN	Fs	ID
	p.Arg564X	c.1690c>T					DN	Sg	ASD+ID
		c.1899+1G>T					DN	Ss	ASD+ID
		c.2024+5G>A					DN	Sg	ID
	p.Glu714X	c.2140G>T					DN	Sg	ASD+ID
	p.Tyr854X	c.2562_2563del_p.					DN	Sg	ASD+ID
	p.Asn873Asp	c.2617A>G					DN	Sg	ASD+ID
	p.Thr976Lys	c.2927C>A					DN	Fs	ID
		c.3011_3012del					DN	Sp	ASD+ID
		c.3518+5G>C					DN	Fs	ASD+ID
		c.3528_3529insAA					Inherited	Fs	ID
		c.3569_3587del					DN	Sg	ID
		c.4093_4094del					DN	Fs	ASD+ID
	p.Arg1472X	c.4414C>T					DN	Sg	ASD+ID
	p.Ser1420X	c.4259_4260del					DN	Fs	ID
		c.5386del					IN	Sg	ID
		c.5599+2T>C					N/A	Fs	ID
		c.6115del					n/a	Fs	ID

		c.7511dup					DN	Fs	ID
Cotney et al. 2015 (18,25)	p.Arg286Cys	c.856C > T						Missense	ASD
	p.Arg2158Cys	c.6472C > T						Missense	ASD
	p.Arg2180Cys	c.6538C > T						Missense	ASD
	p.G2277Ala	c.6830G >C						Missense	ASD
	p.Arg2314Gln	c.6941G > A						Ns	ASD
Siu et al. 2019 (26)	p.Arg1173Gly	c.3517 A>G					DN		ASD
	p.Asn740Ser	c.2219A>G					IN		ASD
	p.Pro2281Ala	c.6841C>G					IN		ASD
	p.Gly2189Arg	c.6565G>A					IN		ASD
	p.Ala1314Thr	c.3940G>A;					IN		ASD
	p.His2500Pro	c.7499A>C					n/a		ASD
	p.Thr2050fs	c.6148dupA					DN		ASD
		c.4215G>T					ID	Sn	ASD
	p.Arg2217	c.6649C>T					ID		ASD
	p.Pro2316LeufsX39	c.6947delC					n/a		ASD
	p.Arg1443Cys	c.4327C>T					ID		ASD
Douzgou et al. 2019 (27)	p.Ser1420X	c.4259_4261GTC>G	21869143-2186914				n/a	Sg	ASD+ID
	p.Glu1004ValfsX22	c.3011_3012GA>T	21873918-21873920				DN	Fs	DD
		c.1899+1G	21883883-21883883				DN	Sp	ASD+ID
	p.Lys545AsnfsX47	c.1635delCA>C	21894367-21894368				n/a	Fs	ASD+ID
	p.Arg1242X	c.3724C>T_	21870653-21870653				DN	Sg	ASD+ID+DD
	p.Gln687X	c.2059C>T	21882543-2188254				DN	Sg	ASD+ID

	p.Tyr902X	c.2706GT>G	21876494-2187649				DN	Sg	ASD
	p.Tyr854X	c.2562_2563del	21876637-21876639				DN	Sg	ASD+ID
		c.3518+1	21877595-21877595				DN	Sp	ID
	p.Leu1206X	c.3617T>G	21871273--21871273				DN	Sg	ASD+ID
Smol et al. 2020 (28)		401 kb microduplication	21499240 - 21899985 × 3					Md	ASD+DD+ID
		277 kb duplication	21622823 - 21899759 × 3						DD
Tran et al. 2020 (29)	p.Ile1192Thr	c.3575 T > C	21871315				DN	Ms	ASD
Wu et al. 2020 (30)	p.G1602Vfs*13	c.4800delA	21399998-21399998	79			DN	Fs	ASD
	p.N885Tfs*14	c.2654delA	21408388-21408388				IN	Fs	ASD
Alotaibi and Ramzan 2020 (31)	p.Arg1662Ter	c.4984C>T					DN	Pms	ASD+DD+ID
An et al. 2020 (32)	p.Tyr1168Asn	c.3502 T>A					DN	Ms	ASD
	p.Arg1188X	c.3562C>T					DN	Ns	ASD+ID
		c.4818-1G>A					DN	Sp	ASD

	p.Glu689X	c.2065C>A					DN	Ns	ASD+DD
	p.Lys2286Arg	c.6857A>G						Ms	ASD
	p.Arg773Gln	c.2318G>A					IN		ASD
	p.Val2521Ala	c.7562T>C					IN		ASD

Abbreviations: **AA change-** amino acid change, **S.M**-sequencing mutation, **Chr14 P-** chromosome 14 position, **NVIQ-** non-verbal I.Q, **VIQ-** verbal I.Q, **FSIQ-** Full Scale I.Q, **INT-** inherited type**, MutT**-mutation type, **Phen-** phenotype. **DN-** de novo, **IN-** inherited, **Fs**-frameshifting indel, **Ns**-nonsense, **Sp-** splice-site, **Aa-** single amino acid deletion, **Ms**-missense, **Mns-** Missense near splice site, **Tr-** Translocation, **Ssv-** Splice site variant, **nSn-** non-synonymous, **Sn-** synonymous, **Sg-** stop-gained, **Pms-** premature stop codon, **Md-** microdeletion, **Dup-** duplication, **LGD-** likely gene-disruptive, **ASD-** autism spectrum disorder, **ID-** intellectual disability, **DD-** developmental delay, **SCZ-** Schizophrenia.

Fs-frameshift, X-stop, X23-stop after 23 amino acid, Ter- stop, Arg 1023del- amino acid Arg in position 1023 was deleted.

References:

1. O'Roak BJ, Vives L, Girirajan S, Karakoc E, Krumm N, Coe BP, et al. Sporadic autism exomes reveal a highly interconnected protein network of de novo mutations. Nature. 2012;485(7397):246–50.

2. O'Roak BJ, Vives L, Fu W, Egertson JD, Stanaway IB, Phelps IG, et al. Multiplex targeted sequencing identifies recurrently mutated genes in autism spectrum disorders. Science (80-) [Internet]. 2012 Dec 21 [cited 2020 Oct 26];338(6114):1619–22. Available from: https://www.sciencemag.org/lookup/doi/10.1126/science.1227764

3. Bernier R, Golzio C, Xiong B, Stessman HA, Coe BP, Penn O, et al. Disruptive CHD8 mutations define a subtype of autism early in development. Cell [Internet]. 2014;158(2):263–76. Available from: http://dx.doi.org/10.1016/j.cell.2014.06.017

4. Prontera P, Ottaviani V, Toccaceli D, Rogaia D, Ardisia C, Romani R, et al. Recurrent ∼100 Kb microdeletion in the chromosomal region 14q11.2, involving CHD8 gene, is associated with autism and macrocephaly. Am J Med Genet Part A. 2014;164(12):3137–41.

5. O'Roak BJ, Stessman HA, Boyle EA, Witherspoon KT, Martin B, Lee C, et al. Recurrent de novo mutations implicate novel genes underlying simplex autism risk. Nat Commun [Internet]. 2014;5. Available from: www.nature.com/naturecommunications

6. Iossifov I, O'Roak BJ, Sanders SJ, Ronemus M, Krumm N, Levy D, et al. The contribution of de novo coding mutations to autism spectrum disorder. Nature [Internet]. 2014;515(7526):216–21. Available from: http://dx.doi.org/10.1038/nature13908

7. De Rubeis S, He X, Goldberg AP, Poultney CS, Samocha K, Cicek AE, et al. Synaptic, transcriptional and chromatin genes disrupted in autism. Nature [Internet]. 2014;515(7526):209–15. Available from: http://dx.doi.org/10.1038/nature13772

8. Talkowski ME, Rosenfeld JA, Blumenthal I, Pillalamarri V, Chiang C, Heilbut A, et al. Sequencing chromosomal abnormalities reveals neurodevelopmental loci that confer risk across diagnostic boundaries. Cell [Internet]. 2012;149(3):525–37. Available from: http://dx.doi.org/10.1016/j.cell.2012.03.028

9. McCarthy SE, Gillis J, Kramer M, Lihm J, Yoon S, Berstein Y, et al. De novo mutations in schizophrenia implicate chromatin remodeling and support a genetic overlap with autism and intellectual disability. Mol Psychiatry [Internet]. 2014;19(6):652–8. Available from: http://www.1000genomes.org/

10. Kimura H, Wang C, Ishizuka K, Xing J, Takasaki Y, Kushima I, et al. Identification of a rare variant in CHD8 that contributes to schizophrenia and autism spectrum disorder susceptibility. Schizophr Res. 2016;178(1–3):104–6.

11. Merner N, Forgeot d'Arc B, Bell SC, Maussion G, Peng H, Gauthier J, et al. A de novo frameshift mutation in chromodomain helicase DNA-binding domain 8 (CHD8): A case report and literature review. Am J Med Genet Part A. 2016;170(5):1225–35.

12. Smyk M, Poluha A, Jaszczuk I, Bartnik M, Bernaciak J, Nowakowska B. Novel 14q11.2 microduplication including the CHD8 and SUPT16H genes associated with developmental delay. Am J Med Genet Part A. 2016;170(5):1325–9.

13. Zahir F, Firth H V, Baross A, Delaney AD, Eydoux P, Gibson WT, et al. Novel deletions of 14q11.2 associated with developmental delay, cognitive impairment and similar minor anomalies in three children. J Med Genet [Internet]. 2007;44(9):556–61. Available from: www.jmedgenet.com

14. Terrone G, Cappuccio G, Genesio R, Esposito A, Fiorentino V, Riccitelli M, et al. A case of 14q11.2 microdeletion with autistic features, severe obesity and facial dysmorphisms suggestive of Wolf-Hirschhorn syndrome. Am J Med Genet Part A. 2014;164(1):190–3.

15. Wang T, Guo H, Xiong B, Stessman HAF, Wu H, Coe BP, et al. De novo genic mutations among a Chinese autism spectrum disorder cohort. Nat

Commun [Internet]. 2016;7. Available from: www.nature.com/naturecommunications

16. Stolerman ES, Smith B, Chaubey A, Jones JR. CHD8 intragenic deletion associated with autism spectrum disorder. Eur J Med Genet [Internet]. 2016;59(4):189–94. Available from: http://dx.doi.org/10.1016/j.ejmg.2016.02.010

17. Han JY, Jang JH, Park J, Lee IG. Targeted next-generation sequencing of Korean patients with developmental delay and/or intellectual disability. Front Pediatr [Internet]. 2018;6:391. Available from: https://www.

18. Wang J, Liu J, Gao Y, Wang K, Jiang K. Autism Spectrum Disorder Early in Development Associated with CHD8 Mutations among Two Chinese Children. BMC Pediatr. 2018;18(1):1–7.

19. D'Gama AMD, Pochareddy S, Li M, Jamuar SS, Reiff RE, Lam AN, et al. Targeted DNA sequencing from autism spectrum disorder brains implicates multiple genetic mechanisms. Neuron. 2015;88(5):910–7.

20. Cappi C, Brentani H, Lima L, Sanders SJ, Zai G, Diniz BJ, et al. Whole-exome sequencing in obsessive-compulsive disorder identifies rare mutations in immunological and neurodevelopmental pathways. Transl Psychiatry. 2016;6(3).

21. Arnett AB, Rhoads CL, Hoekzema K, Turner TN, Gerdts J, Wallace AS, et al. The autism spectrum phenotype in ADNP syndrome. Autism Res. 2018;11(9):1300–10.

22. Wong WR, Brugman KI, Maher S, Oh JY, Howe K, Kato M, et al. Autism-Associated missense genetic variants impact locomotion and neurodevelopment in Caenorhabditis elegans. Hum Mol Genet. 2019;28(13):2271–81.

23. Yasin H, Gibson WT, Langlois S, Stowe RM, Tsang ES, Lee L, et al. A distinct neurodevelopmental syndrome with intellectual disability, autism spectrum disorder, characteristic facies, and macrocephaly is caused by defects in CHD8. J Hum Genet [Internet]. 2019;64(4):271–80. Available from: https://doi.org/10.1038/s10038-019-0561-0

24. Ostrowski PJ, Zachariou A, Loveday C, Beleza-Meireles A, Bertoli M, Dean J, et al. The CHD8 overgrowth syndrome: A detailed evaluation of an emerging overgrowth phenotype in 27 patients. Am J Med Genet Part C Semin Med Genet. 2019;181(4):557–64.

25. Cotney J, Muhle RA, Sanders SJ, Liu L, Willsey AJ, Niu W, et al. The autism-associated chromatin modifier CHD8 regulates other autism risk genes during human neurodevelopment. Nat Commun [Internet]. 2015;6:1–11. Available from: http://dx.doi.org/10.1038/ncomms7404

26. Siu MT, Butcher DT, Turinsky AL, Cytrynbaum C, Stavropoulos DJ, Walker S, et al. Functional DNA methylation signatures for autism spectrum disorder genomic risk loci: 16p11.2 deletions and CHD8 variants. Clin Epigenetics. 2019;11(1):1–19.

27. Douzgou S, Liang HW, Metcalfe K, Somarathi S, Tischkowitz M, Mohamed W, et al. The clinical presentation caused by truncating CHD8 variants. Clin Genet. 2019;96(1):72–84.

28. Smol T, Thuillier C, Boudry-Labis E, Dieux-Coeslier A, Duban-Bedu B, Caumes R, et al. Neurodevelopmental phenotype associated with CHD8-SUPT16H duplication. Neurogenetics. 2020;21(1):67–72.

29. Tran KT, Le VS, Bui HTP, Do DH, Ly HTT, Nguyen HT, et al. Genetic landscape of autism spectrum disorder in Vietnamese children. Sci Rep [Internet]. 2020;10(1):1–11. Available from: http://dx.doi.org/10.1038/s41598-020-61695-8

30. Wu H, Li H, Bai T, Han L, Ou J, Xun G, et al. Phenotype-to-genotype approach reveals head-circumference-associated genes in an autism spectrum disorder cohort. Clin Genet. 2020;97(2):338–46.

31. Alotaibi M, Ramzan K. A de novo variant of CHD8 in a patient with autism spectrum disorder. Discoveries [Internet]. 2020;8(1):e107. Available from: www.discoveriesjournals.org/discoveries

32. An Y, Zhang L, Liu W, Jiang Y, Chen X, Lan X, et al. De novo variants in the Helicase-C domain of CHD8 are associated with severe phenotypes including autism, language disability and overgrowth. Hum Genet. 2020;139(4):499–512.
