# Supplementary material for: The Mechanisms of CHD8 in Neurodevelopment and Autism Spectrum Disorders
Source: Genes (Basel). 2021 Jul 26;12(8):1133. doi: 10.3390/genes12081133 (PMC8393912; doi:10.3390/genes12081133)
Supplement: Supplementary file 1 [file genes-12-01133-s001.zip › supplementary table 2.pdf]

Supplementary Table S2: CHD8 mouse models.

Mouse model	Chd8+/ΔSL and Chd8+/ΔL	Chd8+/-	Chd8+/del5	Chd8+/−	Chd8$^{+/N2373K}$	Chd8V986*/+	Chd8+/E31 T	Olig1-Cre/Chd8F/F mice
Reference	Katayama et al. 2016 (1)	Platt et al. 2017 (2)	Gompers et al. 2017 (3)	Suetterlin et al. 2018 (4)	Jung et al. 2018 (5)	Jiménez et al. 2020 (7)	Hulbert et al. 2020 (8)	Kawamura et al. 2020 (9)
Mutation	Deletion of Exons 12–14 (“ΔL”) And Deletion of Exons 2–10 (“ΔSL”)	A 7-nucleotide deletion in exon 1 that causes a frameshift mutation leading to loss-of-function	Novel germline 5 bp deletion in Chd8 exon 5.	Early frameshift and termination of translation at amino acid 419 at exon 3. Chd8flox/+ crossed with β-actinCre mice to generate β-actinCre;Chd8+/− mice.	Asn2373LysfsX2 in mice parallel to Asn2371LysfsX2 in humans. In exon 37.	Stop codon at the valine 986 which equivalent to valine 984 in mouse Chd8.	Gene trap inserted after Exon 31.	Deletion of Exons 12–14 (“ΔL”) in oligodendrocyte s
Brain volume	Increased (at E18.5 and adult)	n/a	Increased<br><br>Maximal cortical anteroposterior length of Chd8+/del5 brains was ~7% longer at P0 (no substantial differences between sexes), 7.5% increase in absolute volume of cortex, whole-mount and Nissl-stained coronal brain sections at P7- no neuropathologic	Increased<br><br>Total brain increased by 2.7%, several brain regions, including cortical areas, hippocampus and parts of the cerebellum showed volumetric increases	Increase (MRI)	Increased (P0)	n/a	No differ

			al anomalies were observed, cortical thickness at 30% and 70% distance from the dorsal midline- no significant differences. The overall neocortical section area was ~8% larger, cerebral white matter and cerebral gray matter were larger at 5.4% and 6.1% respectively. Robust increases in absolute volume across cortical regions, hippocampus (+10.3%) and amygdala (+11.0%). Increased cortical thickness. Deep cerebellar nuclei showed decreased relative volume (-1 to -3%).					
Brain morphology and function	n/a	Morphological analysis using Nissl staining	Increased absolute volume of cerebral cortex (f-stat =	interorbital distance- significantly wider	anterior cingulate, anterior commissure, and cerebellum in female (not in male)	n/a	length increase for adult mice	number of oligodendrocytes was significantly

(MRI, µCT, rsfMRI)		shows no overt phenotype present in the somatosensory cortex<br><br>no increase in the number of cortical progenitor cells as measured by BrdU incorporation within the somatosensory cortex<br><br>no increase in either the total cell-cycle length or the length of S phase within the somatosensory cortex<br><br>(no gross defects in specification, migration, or lamination of different subtypes in the neocortex.)<br><br>increase in both intraocular	33.6, FDR < 0.1%), hippocampus (f-stat = 29.0, FDR < 0.1%) and amygdala (f-stat = 38.6, FDR < 0.1%)<br><br>increased brain volume<br><br>(MRI)	anterior–posterior length of the interparietal bone – increased, suggestive of more widespread craniofacial anomalies<br><br>(µCT)<br><br>hotspots for increased connectivity in Cortical and Hippocampal Networks (entorhinal, retrosplenial, auditory cortical and posterior hippocampal areas), increased cortical connectivity in auditory regions, increase in connectivity between ventral hippocampus and auditory cortical regions, increased connectivity of this region with both cingulate	Gross brain morphology-normal<br><br>hippocampus displays sexually dimorphic synaptic transmission and neuronal firing<br>(MRI)			reduced in the corpus callosum of Olig1-Cre/Chd8L F/F mice at P7 and P14

		distance as well as total brain volume (10W) (MRI)		and entorhinal cortices<br><br>(rsfMRI)				
Body weight	No differ	Decrease (10W)	No differ	Decrease (5W)	No differ	No differ at P0<br>Decrease at 25W	n/a	No differ
Transcriptomic changes in the brain								
* RNA-seq	n/a	Brain development, epigenome regulation, neuronal and synaptic adhesion.	Forebrain at E12.5, E14.5, E17.5, P0 and adult mice<br><br>Chd8 expression declined across development.<br><br>Decreased in genes of RNA processing, chromatin remodeling, and cell cycle, increased of genes linked to immune function and cell identity of astrocytes or microglia.<br>Alteration of genes involved in neuronal maturation<br><br>Increased prenatal proliferation of neural progenitors,	Neocortical tissue at E12.5 and P5<br><br>Upregulated - KEGG pathways related to protein transport, the ribosome and oxidative phosphorylation<br><br>downregulated genes related to cell adhesion, axonal guidance and calcium signaling pathways, Suz12 targets genes.<br><br>At P5 significant enrichment of cell adhesion and axonal guidance genes in the downregulated genes.<br>axon guidance and cell adhesion genes that are	Hippocampus at P0, P25<br><br>Chd8 expression was stronger than at later stages<br><br>No differentially expressed genes (DEGs) in P0 in males or females.<br>Sexually dimorphic enrichment patterns at P25, there were three DEGs in males and 96 DEGs in females. In P25 females- extracellular vesicles, including blood microparticles and extracellular exosomes, platelet activation.<br><br>P25 female whole-brain enriched genes were 'blood microparticle' and 'platelet'.<br>P25 male, but not female, whole-brain were strongly and negatively enriched genes for 'synapse'.<br>Males and females showed largely similar enrichment patterns for	Cerebral cortex at E14.5, 1M, 6M, 12M<br>Chd8 expression was highest at E14.5 and persisted at a lower level throughout life<br><br>genes associated with focal adhesion, neurodevelopmenta l, proteostasis, sodium channel activity and synaptic function were reduced<br><br>reduced mTORC1 and IRE1 pathway activation<br><br>decreased XBP1 expression<br><br>genes associated with heat shock factor 1 (HSF1) signaling and chaperone function were reduced	n/a	oligodendrocyte -specific genes were down-regulated

				preferentially expressed in CA2 and auditory areas at adult stages and whose expression is dysregulated at P5	‘ribosome’ and ‘mitochondria/oxidoreduc- tase’ genes	c- MET signaling pathways were upregulated<br><br>protein homeostasis is impaired		
* ChIP-seq	Expression of genes related to synapses and ion was downregulated in Chd8+/ΔL mice. Neural development is delayed during the early to mid-fetal stage in the mutant mice	CHD8 binding sites are enriched in promoters. Enrichment for histone and chromatin modification as well as al-terations in mRNAand protein processing.	Strong concordance in enriched functional annotation terms between DE and Chd8-bound genes. Strong enrichment for binding among downregulated genes.	n/a	n/a	n/a	n/a	significant enrichment for myelination, membrane, lipid metabolic process and sterol biosynthesis process.
Repetitive behavior								
*Self-grooming	No differ	No differ	No differ	No differ	Increase in adult male when isolated for 3 d, but showed normal self-grooming and other repetitive behaviors when housed together, whereas females showed no isolation-induced self-grooming	n/a	No differ	
* Marble burying	n/a	No differ	No differ	No differ	No differ	No differ	n/a	
Anxiety like behavior	Increased	Increased	n/a		In adult male- no differ	n/a	No differ	Increased

Learning impairments								
* Light–dark emergence task	Decrease	Increased in the latency to enter the light side	n/a	No differ	n/a	n/a	No differ	n/a
* Elevated plus maze	Decrease	n/a	n/a	n/a	No differ	No differ (6M)	No differ	No differ
* Elevated zero maze	n/a	n/a	n/a	n/a	n/a	n/a	No differ	n/a
* T-maze forced-alternation test	No differ Chd8+/ΔL mice	n/a	n/a	n/a	n/a	n/a	n/a	n/a
* T-maze left–right discrimination test	percentage of correct responses was reduced for Chd8+/ΔL mice	n/a	n/a	n/a	n/a	n/a	n/a	n/a
* Open field	Center time – Decrease<br><br>Total distance- no differ	Center time – Decrease<br><br>Total distance- Decrease<br><br>Reduced locomotion	No differ	Hypoactivity<br><br>No evidence of anxiety<br><br>Total distance- Decrease<br><br>Center time- no differ	Hypoactivity and decreased center time in adult male but not female<br><br>Total distance- decrease<br><br>Center time- decrease	Distance traveled. No significant differences<br><br>Number of rears - significant decrease<br><br>Time in center. No significant differences at 6M but significant decrease in center time at 12M	No differ	No differ
* Acoustic startle test	Amplitude- Decrease<br>Prepulse inhibition- Increase	n/a	n/a	n/a	Prepulse inhibition- no differ	Amplitude - no differ<br>Prepulse inhibition- no differ	n/a	No differ
*Contextual fear conditioning	n/a	No differ	Deficits in learning and memory, less	n/a	n/a	n/a	n/a	n/a

			freezing, lower freezing scores to the auditory cue					
* Tone fear conditioning	n/a	No differ	n/a	n/a	n/a	n/a	n/a	n/a
* Novel object recognition	Decrease	n/a	Deficits in recognition	n/a	n/a	n/a	n/a	n/a
*Morris water maze test (Spatial learning abilities and cognitive flexibilit)	n/a	n/a	n/a	Normal in the learning part, normal cognitive, spatial learning abilities and flexibility.	n/a	n/a	n/a	n/a
* T-maze left-right discrimination test	n/a	n/a	n/a	n/a	n/a	n/a	n/a	No differ
Olfactory	n/a	n/a	n/a	Increased interest in an odour with social significance	in adult male- no differ	No differ	n/a	No differ
Motor function								
* Rotarod	increase in acquired motor learning	n/a	n/a	normal motor abilities	in adult male- no differ	n/a	increase in motor performanc e	n/a
*Forelimb grip strength	n/a	n/a	n/a	slightly but significantly reduced	n/a	n/a	n/a	n/a
Sociability								
Social communicatio n	n/a	Mild deficit in social interaction behavior in	No differ	No obvious communication deficit	n/a	Increased social preference	No differ	Mild deficit in social-novelty preference but

		the social novelty but not the sociability test of the three-chambered social approach task						not in sociability
*Three-chamber test for social novelty preference	Sociability- no differ<br><br>Novelty preference- Decrease	Sociability and Entries- no differ<br><br>Novelty preference- Decrease	No differ	Normal sociability<br><br>minor deficits in social novelty	In adult male- no differ<br><br>Sociability and Novelty preference- no differ	Sociability- no duffer<br>Novelty preference – increased<br>a shift in preference to the newly introduced stranger (6M+12M)<br>Entries-no differ	No differ	Significant preference for a novel mouse
*social-interaction test	reduced for mutant mice<br><br>duration per contact was greatly increased	n/a	n/a	n/a	in adult male- no differ (dyadic social interaction)	n/a	n/a	Social contacts did not differ<br><br>duration per contact and total contact time-increased
* Communication through USVs ultrasonic vocalizations (USVs)	n/a	n/a	No differ	No differ	More frequently, rapidly, and for longer durations in male P5-11 but not in female (when separated from their mothers)<br><br>In adult male- no differ	Increased social interest	No difference in the number of calls made, increased call duration	n/a
Cognitive impairments								
* Operant conditioning task	n/a	n/a	n/a	n/a	n/a	n/a	No differ	n/a

Maternal-homing test	n/a	n/a	n/a	n/a	Spent more time with the reunited mothers, suggesting enhanced mother-attachment behavior in male but not female	n/a	n/a	n/a
Homozygous embryonic lethal	+	+	+	n/a	n/a	+	+	Normal at birth, although died before 3 weeks
Other	*Shorter intestine and tended to manifest slower intestinal transit	*Using CRISPR/Cas 9<br>*Synaptic Dysfunction within MSNs in the NAc<br>*local decrease of inhibitory transmission may contribute to the enhanced excitatory inputs onto MSNs in the NAc<br>*CHD8 expression in adults is not required for the increased anxiety-like or decreased locomotor behavior but is required for acquired motor	*Using CRISPR/Cas9	n/a	*In adult male- no differ- nest building and sleeping (huddling).<br>*Opposite changes in inhibitory synaptic transmission in the male and female Chd8+/N2373K Hippocampus	*Using CRISPR/Cas9<br>* Pup survival at P2- reduced when litters were reared by Chd8V986*/+ dams		*Defective myelin formation<br>*Nest-building test and Porsolt forced-swim test - no differ

		learning in the ro- tarod performance test.						
Reference	Katayama et al. 2016 (1)	Platt et al. 2017 (2)	Gompers et al. 2017 (3)	Suetterlin et al. 2018 (4)	Jung et al. 2018 (5)	Jiménez et al. 2020 (7)	Hulbert et al. 2020 (8)	Kawamura et al. 2020 (9)

Reference:

1. Katayama Y, Nishiyama M, Shoji H, Ohkawa Y, Kawamura A, Sato T, et al. CHD8 haploinsufficiency results in autistic-like phenotypes in mice. Nat Publ Gr [Internet]. 2016;537(7622):675–9. Available from: http://dx.doi.org/10.1038/nature19357

2. Platt RJ, Zhou Y, Slaymaker IM, Shetty AS, Weisbach NR, Kim JA, et al. Chd8 Mutation Leads to Autistic-like Behaviors and Impaired Striatal Circuits. Cell Rep [Internet]. 2017;19(2):335–50. Available from: http://dx.doi.org/10.1016/j.celrep.2017.03.052

3. Gompers AL, Su-Feher L, Ellegood J, Copping NA, Riyadh MA, Stradleigh TW, et al. Germline Chd8 haploinsufficiency alters brain development in mouse. Nat Neurosci. 2017;20(8):1062–73.

4. Suetterlin P, Hurley S, Mohan C, Riegman KLH, Pagani M, Caruso A, et al. Altered neocortical gene expression, brain overgrowth and functional over-connectivity in chd8 haploinsufficient mice. Cereb Cortex. 2018;28(6):2192–206.

5. Jung H, Park H, Choi Y, Kang H, Lee E, Kweon H, et al. Sexually dimorphic behavior, neuronal activity, and gene expression in Chd8-mutant mice. Nat Neurosci [Internet]. 2018;21(9):1218–28. Available from: www.nature.com/natureneuroscience1218

6. Zhao C, Dong C, Frah M, Deng Y, Marie C, Zhang F, et al. Dual Requirement of CHD8 for Chromatin Landscape Establishment and Histone Methyltransferase Recruitment to Promote CNS Myelination and Repair. Dev Cell [Internet]. 2018;45(6):753-768.e8. Available from: https://doi.org/10.1016/j.devcel.2018.05.022

7. Jiménez JA, Ptacek TS, Tuttle AH, Schmid RS, Moy SS, Simon JM, et al. Chd8 haploinsufficiency impairs early brain development and protein homeostasis later in life. Mol Autism. 2020;11(1):1–15.

8. Hulbert SW, Wang X, Gbadegesin SO, Xu Q, Xu X, Jiang YH. A Novel Chd8 Mutant Mouse Displays Altered Ultrasonic Vocalizations and Enhanced Motor Coordination. Autism Res. 2020;13(10):1685–97.

9. Kawamura A, Katayama Y, Nishiyama M, Shoji H, Tokuoka K, Ueta Y, et al. Oligodendrocyte dysfunction due to Chd8 mutation gives rise to behavioral deficits in mice. Hum Mol Genet. 2020;29(8):1274–91.
